# Draft Genome Sequence of “*Candidatus* Liberibacter americanus” Bacterium Associated with Citrus Huanglongbing in Brazil

**DOI:** 10.1128/genomeA.00275-13

**Published:** 2013-05-23

**Authors:** Hong Lin, Helvecio D. Coletta-Filho, Cliff S. Han, Binghai Lou, Edwin L. Civerolo, Marcos A. Machado, Goutam Gupta

**Affiliations:** USDA-ARS San Joaquin Valley Agricultural Sciences Center, Parlier, California, USAa; Instituto Agronômico, Centro de Citricultura Sylvio Moreira, Cordeirópolis, São Paulo, Brazilb; Los Alamos National Laboratory, Los Alamos, New Mexico, USAc; Guangxi Citrus Research Institute, Guilin, Guangxi, Chinad

## Abstract

We report here the draft genome sequence of “*Candidatus* Liberibacter americanus” strain PW_SP. The 1,176,071-bp genome, with 31.6% G+C content, comprises 948 open reading frames, 38 tRNAs, and three complete rRNAs.

## GENOME ANNOUNCEMENT

The genus of “*Candidatus* Liberibacter” belongs to the Gram-negative alphaproteobacteria. There are five pathogenic “*Ca*. Liberibacter” species in this genus. “*Candidatus* Liberibacter asiaticus,” “*Candidatus* Liberibacter africanus,” and “*Candidatus* Liberibacter americanus” are associated with citrus huanglongbing (HLB) (1). “*Candidatus* Liberibacter solanacearum” is associated with potato zebra chip disease and other diseases of solanaceous crops (2). In addition, “*Candidatus* Liberibacter europaeus” is associated with disease-like symptoms similar to those of Scotch broom in New Zealand (3). “*Ca*. Liberibacter asiaticus” and “*Ca*. Liberibacter americanus” were identified in São Paulo State, Brazil, in 2004 and 2005, respectively (4, 5). “*Ca*. Liberibacter americanus” was the prevalent species and occurred in almost 90% of symptomatic trees in citrus orchards compared with “*Ca*. Liberibacter asiaticus” during the first two years of the HLB outbreak in Brazil. However, this situation has gradually reversed during the last five years, with “*Ca*. Liberibacter asiaticus” now being found in the majority of HLB-affected citrus orchards in São Paulo State (6). While the reason for this is not clear, it has been suggested that the heat sensitivity of “*Ca*. Liberibacter americanus” and the high titer in “*Ca*. Liberibacter asiaticus”-infected symptomatic plants might account for a competitive advantage of the latter during the natural transmission by psyllids (6, 7). Hitherto, “*Ca*. Liberibacter americanus” has only been found in Brazil. Both “*Ca*. Liberibacter americanus” and “*Ca*. Liberibacter asiaticus” are transmitted by an insect vector, the Asian citrus psyllid (*Diaphorina citri*) (1).

To gain insight into its bacterial genomic information, we sequenced the genome of “*Ca*. Liberibacter americanus” strain PW_SP from São Paulo, Brazil. Genomic DNA was obtained from dodder-infected periwinkle plants (*Catharanthus roseus*) grown in a screen house at Centro de Citricultura, in Cordeiropolis, São Paulo, Brazil. The draft genome sequence of the “*Ca*. Liberibacter americanus” genome was obtained by an Illumina HiSeq 2000 with a 300-bp paired-end library that achieved 18.6× coverage for the “*Ca*. Liberibacter americanus” genome. *De novo* assembly was performed using the Velvet assembler version 1.1. Twenty-two contigs were identified as belonging to the “*Ca*. Liberibacter americanus” genome via BLASTn and BLASTx analyses against the reference genome of “*Ca*. Liberibacter solanacearum” (accession no. CP002371) (8). All 22 “*Ca*. Liberibacter americanus” contigs were confirmed by PCR. Final annotation was reconfirmed by the NCBI Prokaryotic Genomes Annotation Pipeline (PGAAP) (http://www.ncbi.nlm.nih.gov/genomes/static/Pipeline.html). The “*Ca*. Liberibacter americanus” strain PW_SP genome comprises 1,176,071 nucleotides with a G+C content of 31.6%, 948 predicted coding sequences, 38 tRNAs, 3 complete copies of ribosomal RNA genes (16S, 23S, and 5S) and 213 hypothetical genes. While “*Ca*. Liberibacter americanus” is associated with citrus HLB, comparative genome analyses of the “*Ca*. Liberibacter asiaticus,” “*Ca*. Liberibacter solanacearum,” and “*Ca*. Liberibacter americanus” genomes indicated that the “*Ca*. Liberibacter americanus” genome is more similar to that of “*Ca*. Liberibacter solanacearum” (8) than to that of “*Ca*. Liberibacter asiaticus” (9), with respect to their genomic structure and sequence similarity. Phylogenetic analysis using 52 orthologous genes from pathogenic “*Ca*. Liberibacter asiaticus,” “*Ca*. Liberibacter solanacearum,” and “*Ca*. Liberibacter americanus” species, along with the members of *Rhizobiaceae* and other alphaproteobacteria, shows that “*Ca*. Liberibacter americanus” is closely positioned proximal to the basal node, followed by “*Ca*. Liberibacter solanacearum” and “*Ca*. Liberibacter asiaticus,” indicating an early divergence of “*Ca*. Liberibacter americanus.” An additional “*Ca*. Liberibacter americanus” genome sequence extends comparative genome analyses and advances the understanding of the reductive genome evolution in “*Ca*. Liberibacter.”

### Nucleotide sequence accession number.

The whole-genome sequence of “*Ca*. Liberibacter americanus” strain PW_SP has been deposited in GenBank under the accession no. AOFG00000000.
